# Trajectories in waist circumference and waist-to-height ratio with left ventricular hypertrophy in childhood

**DOI:** 10.3389/fnut.2024.1506191

**Published:** 2024-12-18

**Authors:** Xuli Jin, Min Zhao, Jiahong Sun, Bo Xi

**Affiliations:** ^1^Department of Epidemiology, School of Public Health, Cheeloo College of Medicine, Shandong University, Jinan, Shandong, China; ^2^Department of Nutrition and Food Hygiene, School of Public Health, Cheeloo College of Medicine, Shandong University, Jinan, Shandong, China; ^3^Department of Preventive Medicine, School of Public Health, Guangdong Medical University, Dongguan, Guangdong, China

**Keywords:** left ventricular hypertrophy, childhood, central obesity, waist circumference, waist-to-height ratio

## Abstract

**Background and objective:**

Previous studies have shown positive associations of waist circumference (WC) and waist-to-height ratio (WHtR) with left ventricular hypertrophy (LVH) among children and adolescents. However, most of these studies were cross-sectional or limited to only two time points. We aim to estimate the association of trajectories in WC and WHtR with LVH during childhood.

**Methods:**

Data were from the prospective “Huantai Childhood Cardiovascular Health Cohort Study” conducted from 2017 to 2023 in Huantai County, Zibo City, Shandong Province. Group-based trajectory modeling was used to categorize WC into three groups: low-increasing, moderate-increasing, and high-increasing trajectories. Similarly, WHtR was categorized into three groups: stabilizing, decreasing, and increasing trajectories. Linear and log-binomial regression analyses were used to examine the associations of WC and WHtR trajectories with increased left ventricular mass index (LVMI) and LVH.

**Results:**

A total of 946 children were included, with 51.9% being boys and an average age of 8 years at baseline. After adjustment for potential covariates, children in the high-increasing WC group and the increasing WHtR trajectory group had increased LVMI (*β* = 5.16 g/m^2.7^, 95% confidence interval (CI): 4.37, 5.95 and *β* = 4.91 g/m^2.7^, 95% CI: 4.15, 5.68) and a higher risk of LVH [risk ratio (RR) = 5.84, 95% CI: 3.39, 10.05 and RR = 7.38, 95% CI: 4.14, 13.14] compared to the low-increasing WC group and stabilizing WHtR group, respectively. Interestingly, the moderate-increasing WC and decreasing WHtR trajectory groups still have an increased LVMI (*β* = 2.83 g/m^2.7^, 95% CI: 2.05, 3.61 and *β* = 2.25 g/m^2.7^, 95% CI: 1.50, 3.01) and a higher risk of LVH (RR = 2.04, 95% CI: 1.00, 4.15 and RR = 2.23, 95% CI: 1.06, 4.71) compared to the low-increasing WC group and stabilizing WHtR group, respectively. Similar results were found when stratified by sex.

**Conclusion:**

We found the risk of LVH was not fully eliminated among children with a decreasing WHtR trajectory. These findings underscore the need for early prevention and continuous monitoring of WC and WHtR to help prevent future sub-clinical cardiovascular damage in childhood.

## Introduction

Left ventricular hypertrophy (LVH), an extreme increase in left ventricular mass (LVM), is a significant risk factor and predictor for cardiovascular morbidity and mortality (1, 2). The identification of cardiovascular risk factors associated with LVH is important, particularly for children and adolescents, because early organ damage may already occur in this age group (3, 4). Fortunately, evidence indicates that adverse ventricular remodeling can be reversed with early intervention (5, 6). Therefore, it is necessary to identify the risk factors that contribute to LVH regression, thereby implementing targeted preventive and therapeutic strategies.

Numerous studies have shown that general obesity, defined by body mass index (BMI), was associated with LVH in children and adolescents (7–10). However, BMI fails to effectively distinguish the distribution between fat mass and fat-free mass. Studies have indicated that waist circumference (WC) and waist-to-height ratio (WHtR), as markers of central obesity, were superior to BMI and more closely associated with cardio-metabolic risk (11, 12). Several studies have shown that central obesity, defined by WC or WHtR, is associated with LVH among children and adolescents (13–21). However, most previous research was cross-sectional or limited to only two time points, which may overestimate or underestimate the true associations because participants who experienced significant WC or WHtR changes over time may eventually return to their initial status. Therefore, it is necessary to capture the full changes in trajectories of WC or WHtR to better understand their associations with LVH, ultimately leading to more effective and targeted interventions.

In this study, we aimed to clarify the association between different trajectories of WC or WHtR measured at four time points and the risk of LVH based on a population-based prospective study. In addition, we aim to explore the effects of gender differences in WC or WHtR trajectories on cardiac damage.

## Methods

Data were from the prospective “Huantai Childhood Cardiovascular Health Cohort Study” conducted in Huantai County, Zibo City, Shandong Province, China (22). Participants were recruited in a large local primary school using a convenient cluster sampling design. The baseline survey was conducted between November 2017 and January 2018 where 1,515 children aged 6–11 years were recruited at baseline and followed up every 2 years (i.e., 2017, 2019, 2021, and 2023). After excluding children and adolescents with missing information on anthropometric measurements, lifestyle behaviors, blood biochemical markers, ultrasound measurements, or WC/WHtR measurements at any time points, 946 children were finally included in the study (Supplementary Figure S1). Supplementary Table S1 presents a comparison of the baseline characteristics between included and excluded participants. The study was approved by the Ethics Committee of the School of Public Health, Shandong University (Approval No. 20160308). All participants voluntarily joined the study, with informed consent provided by their guardians.

Height, weight, and WC were measured twice using standardized calibrated instruments, and the mean values were used for analysis. Blood pressure (BP, HEM 7012, Omron, Osaka, Japan) was measured three times, with differences of less than 10 mmHg between any two measurements, and the mean values of the last two readings were used for data analysis. These methods have been reported in detail previously (22). BMI (kg/m^2^) was calculated as weight (kg) divided by height (m) squared. WHtR was calculated as WC (cm) divided by height (cm). We categorize WC into three groups: low-increasing, moderate-increasing, and high-increasing trajectories. Similarly, we classified WHtR into three groups: stabilizing, decreasing, and increasing trajectories.

A structured self-reported questionnaire was used to collect data on demographics (e.g., age and sex), pubertal development (e.g., menarche for girls or spermatogenesis for boys), and lifestyle behaviors (e.g., intake of fruits and vegetables, intake of carbonated beverages, physical activity (PA), screen time, and sleep duration). The questionnaires were carried out in classrooms by investigators with standardized training, and respondents were personally informed by instructions. The forms were filled out by parents who assisted the children and once completed, they were independently reviewed and verified by two staff members. To ensure reliability, a random selection of 13 students retested the questionnaires 1 week later. The results demonstrated strong consistency between the initial and retested measurements, as evidenced by kappa values greater than 0.8 for each item related to dietary condition (22). The pubertal development variable was categorized as “yes” if boys experienced seminal emission or girls experienced menstruation, and “no” if they did not. Insufficient intake of fruits/vegetables was defined as consuming less than 5 servings per day (23). Frequent intake of carbonated beverages was defined as consuming more than once per week (23). Insufficient PA was defined as less than 1 h of combined vigorous and moderate exercise per day (24). Excessive screen time was defined as more than 2 h of screen exposure per day (25). Insufficient sleep was defined as less than 9 h (6–12 years old) or less than 8 h (13–18 years old) per day (26). An automatic analyzer (Beckman Coulter, AU480) was used to measure blood biochemical markers [e.g., glucose (GLU), triglyceride (TG), total cholesterol (TC), low-density lipoprotein cholesterol (LDL-C), and high-density lipoprotein cholesterol (HDL-C)].

Before the study, 20 subjects were selected and their measurements were taken by two sonographers: one for quality control and the other for testing. The correlation coefficient of the measured values was 0.94, demonstrating a high level of agreement (27). During the formal study, left ventricular indicators were measured using a validated and calibrated color Doppler ultrasound machine (Philips, CX30, United States) by one experienced sonographer. All procedures strictly adhered to the Pediatric Cardiac Measurement Guidelines of the American Society of Echocardiography (28). The left ventricular end-diastolic diameter (LVDD), interventricular septal thickness (IVST), and left ventricular posterior wall thickness (LVPWT) were assessed using a 2e4 MHz convex array transducer. Based on these measurements and height, the LVMI was calculated using the following formulas (16): LVM (g) = 0.8*1.04*[(IVST+LVDD+LVPWT)^3^ − (LVDD)^3^] + 0.6; LVMI (g/m^2.7^) = LVM/height^2.7^. LVH was determined as the LVMI higher than the age- and sex-specific 90th percentile of this study population (10, 29).

*T*-tests and *χ*^2^-tests were, respectively, used to assess the differences in continuous and categorical variables between boys and girls at baseline. Group-based trajectory modeling (GBTM) was used to analyze the trajectories of WC and WHtR with the optimal number of groups determined based on the Bayesian information criterion (BIC), average posterior probability (AvePP), and the proportion of individuals in each trajectory group. Analysis of variance (ANOVA) and *χ*^2^-test were used to assess the differences in continuous and categorical variables across trajectory groups, respectively. We used linear regression to examine the associations of WC or WHtR trajectories from 2017 to 2023 with LVMI at the end of follow-up in three models and used log-binomial regression to assess their associations with LVH. Model 1 adjusted for age, sex, and height (only for WC); Model 2 further adjusted for trajectory groups of systolic BP (SBP), diastolic BP (DBP), GLU, HDL-C, LDL-C, TG, and TC from baseline to follow-up periods; Model 3 additionally adjusted for pubertal development at follow-up, intake of fruits/vegetables, intake of carbonated beverages, PA, screen time, and sleep time at baseline. A two-sided *p* value <0.05 indicated statistical significance. All statistical analyses were performed using R (version 4.3.3).

## Results

In this study, a total of 946 children were included, with 51.9% being boys and an average age of 8 years at baseline (Table 1). At baseline, boys had higher values of BMI, WC, WHtR, SBP, GLU, HDL-C, insufficient intake of fruits/vegetables, frequent intake of carbonated beverages, and LVMI compared to girls (all *p* values <0.05).

**Table 1:** Basic characteristics between the study population at baseline.

Characteristics	Overall (*n* = 946)	Boys (*n* = 491)	Girls (*n* = 455)	*t*/*χ*^2^ value	*p* value
Age, years	8.0 (1.4)	8.0 (1.4)	8.0 (1.5)	−0.13	0.893
BMI, kg/m^3^	17.8 (3.1)	18.2 (3.2)	17.4 (3.0)	4.02	<0.001
WC, cm	61.8 (9.0)	63.5 (9.4)	60.0 (8.2)	5.22	<0.001
WHtR	0.4 (0.0)	0.4 (0.0)	0.4 (0.0)	3.52	<0.001
SBP, mmHg	105.9 (9.1)	106.7 (8.6)	105.1 (9.6)	0.33	0.009
DBP, mmHg	63.4 (6.5)	63.0 (6.3)	63.8 (6.7)	−2.24	0.049
GLU, mmol/L	4.6 (0.5)	4.7 (0.5)	4.5 (0.5)	1.47	<0.001
HDL-C, mmol/L	1.5 (0.3)	1.6 (0.4)	1.5 (0.3)	6.11	<0.001
LDL-C, mmol/L	2.2 (0.6)	2.2 (0.6)	2.2 (0.6)	6.49	0.746
TG, mmol/L	0.7 (0.3)	0.7 (0.3)	0.7 (0.3)	2.59	0.026
TC, mmol/L	4.1 (0.8)	4.1 (0.8)	4.0 (0.7)	−1.97	0.143
Insufficient intake of fruits/vegetables	784 (82.9)	425 (86.6)	359 (78.9)	9.22	0.002
Frequent intake of carbonated beverages	58 (6.1)	39 (7.9)	19 (4.2)	5.19	0.023
Insufficient PA	801 (84.7)	410 (83.5)	391 (85.9)	0.90	0.344
Excessive screen time	39 (4.1)	23 (4.7)	16 (3.5)	0.55	0.460
Insufficient sleep	145 (15.3)	69 (14.1)	76 (16.7)	1.08	0.298
LVMI, g/m^2.7^	28.0 (4.2)	29.0 (4.2)	26.9 (4.1)	7.71	<0.001

Continuous variables are expressed as mean (standard deviation) and categorical variables are expressed as *n* (%). BMI, body mass index; DBP, diastolic blood pressure; GLU, glucose; HDL-C, high-density lipoprotein cholesterol; LDL-C, low-density lipoprotein cholesterol; LVMI, left ventricular mass index; PA, physical activity; SBP, systolic blood pressure; TC, total cholesterol; TG, triglyceride; WC, waist circumference; WHtR, waist-to-height ratio.

**Figure 1:**
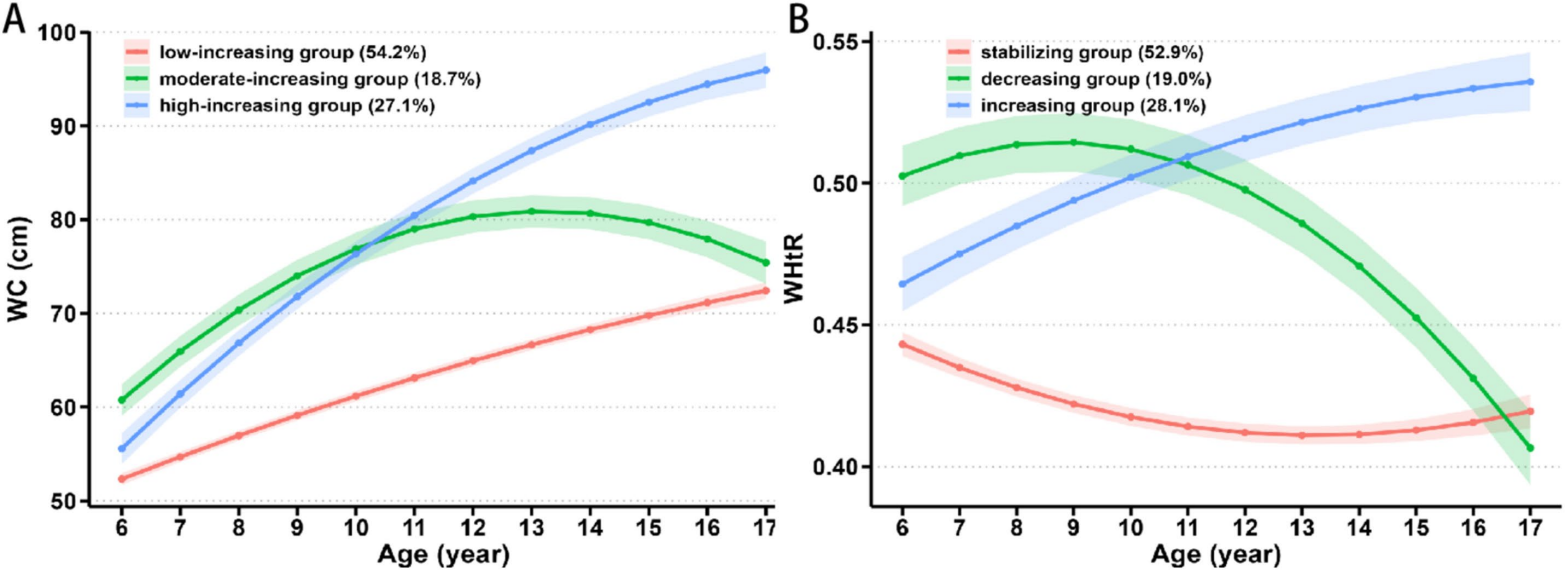
Change in trajectories of **(A)** WC and **(B)** WHtR with age. WC, waist circumference; WHtR, waist-to-height ratio.

Supplementary Table S2 presents the details of the GBTM of trajectory groupings. Based on the BIC, the proportion of AvePP>0.7, and the proportion of individuals in each trajectory group, we selected a three-trajectory grouping for both WC and WHtR. From 2017 to 2023, 54.2, 18.7, and 27.1% of children exhibited low-increasing, moderate-increasing, and high-increasing WC trajectory patterns, respectively (Figure 1A). The moderate-increasing and high-increasing WC trajectory groups had a significantly higher proportion of boys, as well as higher baseline values of BMI, WC, WHtR, SBP, DBP, GLU, LDL-C, TG, LVMI, and LVH compared to the low-increasing group (all *p* values <0.05, Table 2). During the same period, 52.9, 19.0, and 28.1% of children exhibited stabilizing, decreasing, and increasing WHtR trajectory patterns, respectively (Figure 1B). The decreasing and increasing WHtR groups had a significantly higher proportion of boys, as well as higher baseline values of BMI, WC, WHtR, SBP, DBP, GLU, LDL-C, TG, LVMI, and LVH compared to the stabilizing group (all *p* values <0.05, Table 3). At the end of follow-up, similar differences in characteristics across trajectory groups were found (Tables 2, 3).

**Table 2:** Characteristics of baseline (2017) and follow-up (2023) periods of study participants in different WC trajectory subgroups.

Trajectory groups of WC	Low-increasing group (*n* = 513)	Moderate-increasing group (*n* = 177)	High-increasing group (*n* = 256)	F/*χ*^2^ value	*p* value
Baseline (2017)					
Boys	215 (41.9)	95 (53.7)	181 (70.7)	56.99	<0.001
Age, years	8.0 (1.4)	7.8 (1.4)	8.1 (1.5)	1.61	0.200
BMI, kg/m^2^	15.9 (1.6)	20.6 (2.6)	19.8 (3.2)	379.60	<0.001
WC, cm	56.5 (4.7)	69.8 (7.4)	66.9 (9.7)	332.25	<0.001
WHtR	0.4 (0.0)	0.5 (0.0)	0.4 (0.0)	391.88	<0.001
SBP, mmHg	104.1 (9.0)	108.7 (9.3)	107.8 (8.5)	25.52	<0.001
DBP, mmHg	62.2 (6.2)	65.6 (6.3)	64.4 (6.9)	22.56	<0.001
GLU, mmol/L	4.6 (0.5)	4.7 (0.5)	4.7 (0.5)	8.53	<0.001
HDL-C, mmol/L	1.6 (0.3)	1.4 (0.3)	1.5 (0.4)	27.75	<0.001
LDL-C, mmol/L	2.2 (0.6)	2.4 (0.8)	2.3 (0.6)	7.10	0.001
TG, mmol/L	0.6 (0.2)	0.8 (0.4)	0.7 (0.3)	31.52	<0.001
TC, mmol/L	4.0 (0.7)	4.1 (0.9)	4.1 (0.8)	2.26	0.105
Insufficient intake of fruits/vegetables	417 (81.3)	148 (83.6)	219 (85.5)	2.27	0.322
Frequent intake of carbon	29 (5.7)	13 (7.3)	16 (6.2)	0.66	0.718
Insufficient PA	436 (85.0)	148 (83.6)	217 (84.8)	0.19	0.908
Excessive screen time	18 (3.5)	9 (5.1)	12 (4.7)	1.11	0.574
Insufficient sleep	89 (17.3)	21 (11.9)	35 (13.7)	3.79	0.150
LVMI, g/m^2.7^	27.0 (3.8)	29.0 (4.3)	29.2 (4.7)	28.39	<0.001
LVH	21 (4.1)	22 (12.6)	33 (13.0)	23.84	<0.001
Follow-up (2023)					
SBP, mmHg	110.8 (9.9)	119.4 (11.6)	124.2 (11.9)	139.71	<0.001
DBP, mmHg	60.6 (6.8)	61.9 (7.9)	62.8 (7.2)	8.68	<0.001
GLU, mmol/L	4.6 (0.4)	4.6 (0.4)	4.8 (0.4)	7.12	0.001
HDL-C, mmol/L	1.5 (0.2)	1.4 (0.2)	1.3 (0.2)	78.11	<0.001
LDL-C, mmol/L	2.0 (0.6)	2.2 (0.7)	2.3 (0.6)	14.07	<0.001
TG, mmol/L	0.8 (0.3)	0.9 (0.3)	1.0 (0.5)	32.65	<0.001
TC, mmol/L	4.1 (0.7)	4.0 (0.6)	4.1 (0.6)	0.41	0.662
WC, cm	67.8 (5.2)	80.4 (7.1)	89.8 (9.1)	929.25	<0.001
WHtR	0.4 (0.0)	0.4 (0.0)	0.5 (0.0)	773.42	<0.001
LVMI, g/m^2.7^	29.6 (4.0)	32.5 (4.2)	35.3 (5.2)	144.54	<0.001
LVH	20 (3.9)	14 (7.9)	62 (24.1)	78.08	<0.001

Continuous variables are expressed as mean (standard deviation) and categorical variables are expressed as *n* (%). BMI, body mass index; DBP, diastolic blood pressure; GLU, glucose; HDL-C, high-density lipoprotein cholesterol; LDL-C, low-density lipoprotein cholesterol; LVH, left ventricular hypertrophy; LVMI, left ventricular mass index; PA, physical activity; SBP, systolic blood pressure; TC, total cholesterol; TG, triglyceride; WC, waist circumference; WHtR, waist-to-height ratio.

**Table 3:** Characteristics of baseline (2017) and follow-up (2023) periods of study participants in different WHtR trajectory subgroups.

Trajectory groups of WHtR	Stabilizing group (*n* = 500)	Decreasing group (*n* = 180)	Increasing group (*n* = 266)	F/*χ*^2^ value	*p* value
Baseline (2017)					
Boys	216 (43.2)	115 (63.9)	160 (60.2)	32.78	<0.001
Age, years	8.1 (1.4)	7.7 (1.4)	8.1 (1.5)	4.46	0.012
BMI, kg/m^2^	15.9 (1.5)	20.5 (2.6)	19.7 (3.2)	367.98	<0.001
WC, cm	56.6 (4.8)	69.5 (7.8)	66.4 (9.6)	300.10	<0.001
WHtR	0.4 (0.0)	0.5 (0.0)	0.4 (0.0)	449.70	<0.001
SBP, mmHg	104.1 (8.9)	108.3 (9.2)	107.8 (8.8)	22.48	<0.001
DBP, mmHg	62.1 (6.2)	65.3 (6.3)	64.4 (6.9)	21.05	<0.001
GLU, mmol/L	4.6 (0.5)	4.7 (0.5)	4.7 (0.5)	6.41	0.002
HDL-C, mmol/L	1.6 (0.3)	1.4 (0.3)	1.5 (0.4)	28.44	<0.001
LDL-C, mmol/L	2.2 (0.6)	2.4 (0.7)	2.2 (0.6)	5.05	0.007
TG, mmol/L	0.6 (0.2)	0.8 (0.3)	0.7 (0.3)	28.82	<0.001
TC, mmol/L	4.0 (0.8)	4.1 (0.8)	4.1 (0.7)	1.08	0.340
Insufficient intake of fruits/vegetables	405 (81.0)	153 (85.0)	226 (85.0)	2.63	0.269
Frequent intake of carbon	27 (5.4)	16 (8.9)	15 (5.6)	2.95	0.228
Insufficient PA	422 (84.4)	152 (84.4)	227 (85.3)	0.13	0.939
Excessive screen time	18 (3.6)	7 (3.9)	14 (5.3)	1.25	0.536
Insufficient sleep	87 (17.4)	19 (10.6)	39 (14.7)	4.90	0.086
LVMI, g/m^2.7^	26.9 (3.8)	29.8 (4.1)	28.8 (4.6)	39.83	<0.001
LVH	16 (3.2)	30 (16.9)	30 (11.4)	38.14	<0.001
Follow-up (2023)					
SBP, mmHg	111.1 (10.1)	119.4 (11.8)	123.1 (12.1)	113.20	<0.001
DBP, mmHg	60.8 (6.9)	60.9 (8.0)	63.0 (7.0)	9.05	<0.001
GLU, mmol/L	4.6 (0.4)	4.7 (0.4)	4.7 (0.4)	4.94	0.007
HDL-C, mmol/L	1.5 (0.2)	1.4 (0.2)	1.3 (0.2)	74.36	<0.001
LDL-C, mmol/L	2.0 (0.6)	2.1 (0.6)	2.3 (0.6)	14.03	<0.001
TG, mmol/L	0.8 (0.3)	0.8 (0.3)	1.0 (0.5)	37.11	<0.001
TC, mmol/L	4.1 (0.7)	4.0 (0.6)	4.1 (0.6)	2.37	0.094
WC, cm	67.8 (5.3)	80.6 (8.7)	88.6 (9.5)	716.31	<0.001
WHtR	0.4 (0.0)	0.4 (0.0)	0.5 (0.0)	793.42	<0.001
LVMI, g/m^2.7^	29.4 (3.8)	32.6 (4.4)	35.2 (5.1)	157.43	<0.001
LVH	14 (2.8)	13 (7.2)	69 (25.8)	103.52	<0.001

Continuous variables are expressed as mean (standard deviation) and categorical variables are expressed as *n* (%). BMI, body mass index; DBP, diastolic blood pressure; GLU, glucose; HDL-C, high-density lipoprotein cholesterol; LDL-C, low-density lipoprotein cholesterol; LVH, left ventricular hypertrophy; LVMI, left ventricular mass index; PA, physical activity; SBP, systolic blood pressure; TC, total cholesterol; TG, triglyceride; WC, waist circumference; WHtR, waist-to-height ratio.

Children in the moderate-increasing and high-increasing WC trajectory groups had a higher LVMI [*β* = 2.83 g/m^2.7^, 95% confidence interval (CI): 2.05, 3.61 and *β* = 5.16 g/m^2.7^, 95% CI: 4.37, 5.95] and a higher risk of LVH [risk ratio (RR) = 2.04, 95% CI: 1.00, 4.15 and RR = 5.84, 95% CI: 3.39, 10.05] compared to the low-increasing group (Table 4). Similarly, in the WHtR trajectory groups, the decreasing group and increasing group had a higher LVMI (*β* = 2.25 g/m^2.7^, 95% CI: 1.50, 3.01 and *β* = 4.91 g/m^2.7^, 95% CI: 4.15, 5.68) and a higher risk of LVH (RR = 2.23, 95% CI: 1.06, 4.71 and RR = 7.38, 95% CI: 4.14, 13.14) compared to the stabilizing group. Similar results were found when stratified by gender (almost all *p* values <0.05, Table 4).

**Table 4:** Associations of different WC and WHtR trajectory subgroups with LVMI and LVH in children.

Trajectory groups	Model 1		Model 2		Model 3	
	*β*/RR (95% CI)	*p* value	*β*/RR (95% CI)	*p* value	*β*/RR (95% CI)	*p* value
Trajectory groups of WC						
Both						
LVMI^a^, g/m^2.7^						
Low-increasing group	Reference		Reference		Reference	
Moderate-increasing group	3.52 (2.77, 4.27)	<0.001	2.81 (2.04, 3.59)	<0.001	2.83 (2.05, 3.61)	<0.001
High-increasing group	6.08 (5.34, 6.82)	<0.001	5.18 (4.38, 5.97)	<0.001	5.16 (4.37, 5.95)	<0.001
LVH^b^						
Low-increasing group	Reference		Reference		Reference	
Moderate-increasing group	2.61 (1.32, 5.14)	<0.01	1.98 (0.98, 4.00)	0.058	2.04 (1.00, 4.15)	0.051
High-increasing group	8.26 (5.13, 13.29)	<0.001	5.52 (3.23, 9.45)	<0.001	5.84 (3.39, 10.05)	<0.001
Boys						
LVMI^a^, g/m^2.7^						
Low-increasing group	Reference		Reference		Reference	
Moderate-increasing group	3.39 (2.33, 4.45)	<0.001	2.34 (1.32, 3.36)	<0.001	2.35 (1.33, 3.37)	<0.001
High-increasing group	6.23 (5.30, 7.15)	<0.001	5.00 (4.00, 6.01)	<0.001	4.98 (3.97, 5.99)	<0.001
LVH^b^						
Low-increasing group	Reference		Reference		Reference	
Moderate-increasing group	1.91 (0.47, 7.74)	0.365	1.20 (0.34, 4.29)	0.778	1.11 (0.27, 4.52)	0.882
High-increasing group	11.15 (4.66, 26.67)	<0.001	5.65 (2.13, 14.95)	<0.01	6.14 (2.34, 16.14)	<0.001
Girls						
LVMI^a^, g/m^2.7^						
Low-increasing group	Reference		Reference		Reference	
Moderate-increasing group	3.89 (2.83, 4.94)	<0.001	3.49 (2.36, 4.63)	<0.001	3.47 (2.33, 4.60)	<0.001
High-increasing group	5.72 (4.48, 6.96)	<0.001	4.83 (3.52, 6.15)	<0.001	4.82 (3.51, 6.13)	<0.001
LVH^b^						
Low-increasing group	Reference		Reference		Reference	
Moderate-increasing group	2.80 (1.28, 6.11)	<0.05	2.59 (1.11, 6.04)	<0.05	2.70 (1.14, 6.38)	<0.05
High-increasing group	6.52 (3.57, 11.92)	<0.001	4.92 (2.47, 9.81)	<0.001	5.28 (2.64, 10.56)	<0.001
Trajectory groups of WHtR						
Both						
LVMI^a^, g/m^2.7^						
Stabilizing group	Reference		Reference		Reference	
Decreasing group	2.92 (2.18, 3.65)	<0.001	2.22 (1.46, 2.98)	<0.001	2.25 (1.50, 3.01)	<0.001
Increasing group	5.71 (5.01, 6.42)	<0.001	4.95 (4.18, 5.72)	<0.001	4.91 (4.15, 5.68)	<0.001
LVH^b^						
Stabilizing group	Reference		Reference		Reference	
Decreasing group	2.75 (1.31, 5.77)	<0.01	2.15 (1.03, 4.53)	<0.05	2.23 (1.06, 4.71)	<0.05
Increasing group	9.91 (5.71, 17.19)	<0.001	7.23 (4.05, 12.91)	<0.001	7.38 (4.14, 13.14)	<0.001
Boys						
LVMI^a^, g/m^2.7^						
Stabilizing group	Reference		Reference		Reference	
Decreasing group	2.89 (1.94, 3.84)	<0.001	1.85 (0.89, 2.81)	<0.001	1.94 (0.99, 2.89)	<0.001
Increasing group	5.72 (4.75, 6.69)	<0.001	4.51 (3.43, 5.60)	<0.001	4.48 (3.39, 5.58)	<0.001
LVH^b^						
Stabilizing group	Reference		Reference		Reference	
Decreasing group	2.82 (0.81, 9.84)	0.104	1.77 (0.53, 5.91)	0.355	1.76 (0.50, 6.17)	0.375
Increasing group	12.41 (4.49, 34.30)	<0.001	6.84 (2.25, 20.77)	<0.01	7.46 (2.45, 22.76)	<0.001
Girls						
LVMI^a^, g/m^2.7^						
Stabilizing group	Reference		Reference		Reference	
Decreasing group	3.07 (1.92, 4.23)	<0.001	2.76 (1.55, 3.96)	<0.001	2.70 (1.49, 3.91)	<0.001
Increasing group	5.64 (4.62, 6.66)	<0.001	5.11 (4.00, 6.22)	<0.001	5.08 (3.97, 6.18)	<0.001
LVH^b^						
Stabilizing group	Reference		Reference		Reference	
Decreasing group	2.88 (1.12, 7.44)	<0.05	2.79 (1.09, 7.11)	<0.05	2.90 (1.13, 7.47)	<0.05
Increasing group	8.78 (4.53, 17.03)	<0.001	7.46 (3.76, 14.80)	<0.001	7.56 (3.83, 14.92)	<0.001

Model 1: Adjusted for age, sex, and height (only for WC).

Model 2: Model 1 plus trajectory groups of SBP, DBP, GLU, HDL-C, LDL-C, TG, and TC from baseline to follow-up periods.

Model 3: Model 2 plus pubertal development at follow-up, intake of fruits/vegetables, carbonated beverages, PA, screen time, and sleep time at baseline.

DBP, diastolic blood pressure; GLU, glucose; HDL-C, high-density lipoprotein cholesterol; LDL-C, low-density lipoprotein cholesterol; LVH, left ventricular hypertrophy; LVMI, left ventricular mass index; PA, physical activity; RR, risk ratio; SBP, systolic blood pressure; TC, total cholesterol; TG, triglyceride; WC, waist circumference; WHtR, waist-to-height ratio.

a Represents linear regression for continuous variables (*β* values).

b Represents log-binomial regression for categorical variables (RR values).

## Discussion

To the best of our knowledge, this is the first study to estimate the association of WC and WHtR trajectories with LVH during childhood. We found that rapid increases in either WC or WHtR during childhood significantly increase the subsequent risk of LVH. However, the risk of LVH was not fully eliminated among children who had a decreasing WHtR trajectory. These findings not only enrich the current understanding of the impact of childhood central obesity on cardiac damage but also aid in the precise identification of high-risk individuals, thereby enabling more targeted prevention strategies.

Previous studies have evaluated the association between central obesity and LVMI (13, 15–17, 19, 21, 30). For example, an observational study of Italian children aged 7–16 years (*n* = 63) showed that high WC and WHtR were associated with an increased risk of LVH (13). Similarly, another Italian cohort study of 459 obese children with a mean age of 10.6 years demonstrated a positive association between WHtR and higher LVMI (15). Our previous two-year follow-up study on Chinese children aged 6–11 years also demonstrated that persistently high WC at baseline and follow-up had higher LVMI and higher odds of LVH (16). However, prior studies have been cross-sectional or limited to two time points, often limited by small sample sizes and inadequate control of covariates such as dietary habits, PA, changes in blood lipids, and blood glucose, on fluctuations in WC or WHtR, potentially leading to overestimation or underestimation of the true associations (6, 31). Therefore, prospective studies with multiple time points and adequate control of covariates are needed to better understand the temporal changes of central obesity and the risk of cardiac damage.

In this study, we examined the association between trajectory changes in WC or WHtR during childhood with four time points and subsequent LVH with the adjustment of full covariates. As expected (16, 32, 33), we found that both the high-increasing WC and the increasing WHtR trajectory groups were associated with a higher risk of LVH. However, we observed that the risk of LVH was reduced but not fully eliminated in the moderate-increasing WC and the decreasing WHtR trajectory groups, regardless of sex. Two prospective longitudinal cohort studies similarly showed that a moderate increase in WC and a decrease in WHtR during childhood were associated with an increased risk of carotid intima-media thickness and Type-2 diabetes in adulthood (34, 35). Inconsistent with this study, a previous two-year follow-up study showed that the increased odds of LVH could be eliminated among children who had high WC but achieved normal WC 2 years later (16). A 12-month intervention study of 86 children and adolescents aged 5–17 years with essential hypertension found that reductions in WHtR and WC predicted a decrease in LVMI (36). A cohort study of 2,898 Portuguese adolescents showed that decreasing WC trajectory from 13 to 21 years was associated with increased risk of SBP, DBP, insulin resistance, and TG among males, but not females (37). This discrepancy may be mainly attributed to differences in population, outcomes, and classifications of WC or WHtR (i.e., the previous study classified central obesity versus normal status based on two time points, which might have ignored weight regain vs. reduced WC trajectories in this study). Additionally, factors such as dietary and PA habits, as well as changes in blood lipids, glucose, and BP, might affect LVH (38). After adjusting for these covariates, we still found an increased risk of LVH among those with a moderate-increasing WC trajectory or a decreasing WHtR trajectory. These suggest that an increase in WC or WHtR can independently increase the risk of LVH among children and persist even if these measures are later reduced. These findings emphasize the importance of maintaining a healthy WC from an early age to prevent the development of sub-clinical cardiovascular damage. Adopting a healthy diet, increasing PA, reducing sedentary time, and ensuring adequate sleep can aid in maintaining a healthy WC (39, 40).

To the best of our knowledge, this is the first study examining the association of WC and WHtR trajectories with LVMI in childhood. However, several limitations must be acknowledged. First, it used convenience sampling by selecting students from a specific elementary school, therefore limiting the generalizability of the findings. Second, the influence of unmeasured confounders, such as family history of cardiovascular disease and air pollutants might have influenced both adiposity and cardiac structural changes over time. However, the aforementioned and other unmeasured confounders could have attenuated the associations between adiposity and cardiac structural changes toward the null. Third, covariates such as lifestyle behaviors were obtained through self-report, potentially introducing information bias. Fourth, the analysis was limited to gender stratification due to the small sample size.

In conclusion, this study demonstrated that rapid growth in WC and WHtR during childhood can predict subsequent LVH, and the risk persists even if WC and WHtR are later reduced or reversed. These findings underscore the need for early prevention and continuous monitoring of WC and WHtR, along with long-term management of central obesity in children, to help prevent future sub-clinical cardiovascular damage.

## Data Availability

The raw data supporting the conclusions of this article will be made available by the authors, without undue reservation.

## Supplementary material

SUPPLEMENTARY FIGURE S1 Study flowchart. WC, waist circumference; WHtR, waist-to-height ratio.
