# Supplementary figures and images for: Trajectories in waist circumference and waist-to-height ratio with left ventricular hypertrophy in childhood
Source: Front Nutr. 2024 Dec 18;11:1506191. doi: 10.3389/fnut.2024.1506191 (PMC11688229; doi:10.3389/fnut.2024.1506191)

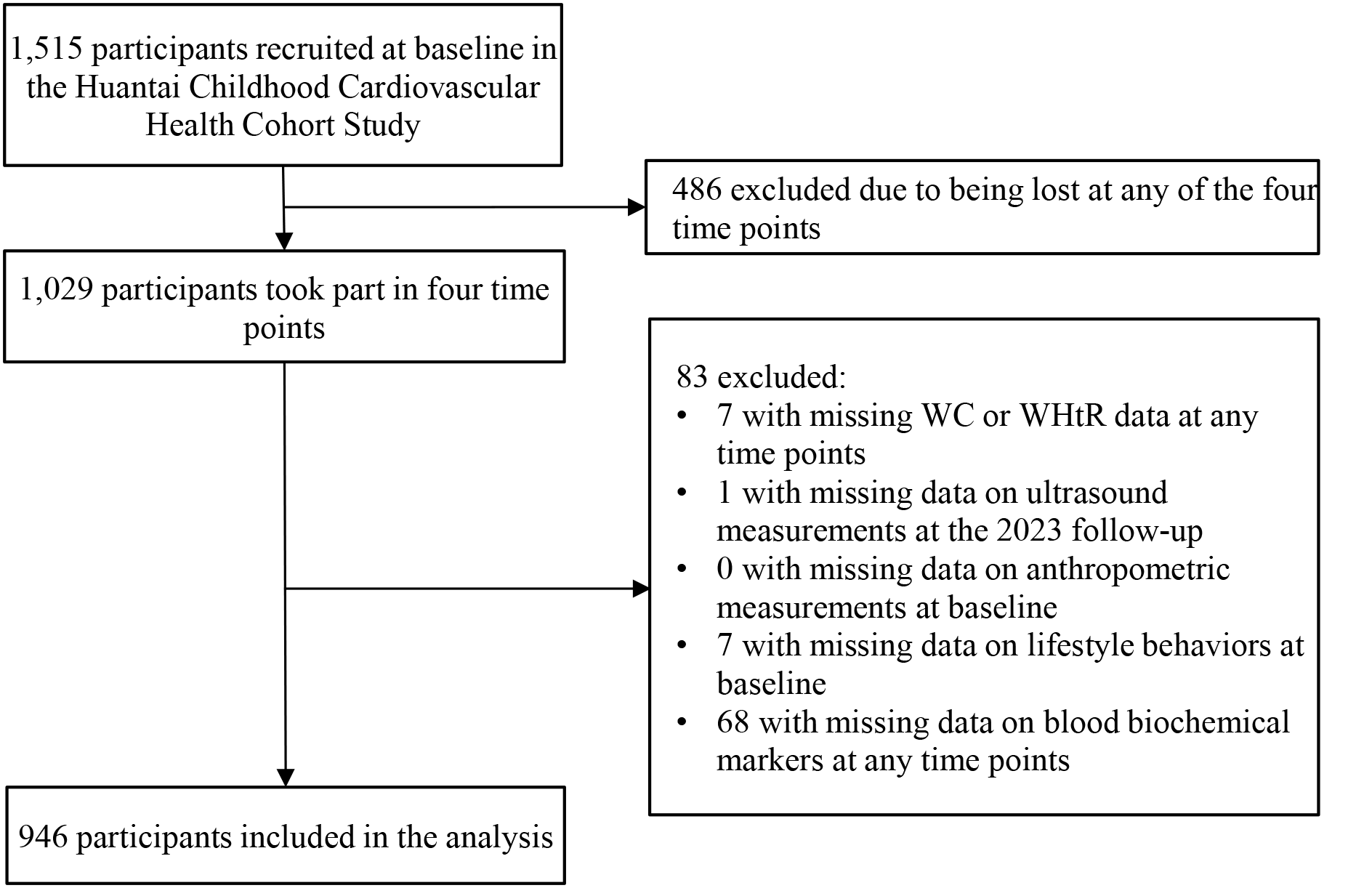

Supplement: SUPPLEMENTARY FIGURE S1 — Study flowchart. WC, waist circumference; WHtR, waist-to-height ratio. [file Image_1.TIF]
