# Supplementary material for: Trajectories in waist circumference and waist-to-height ratio with left ventricular hypertrophy in childhood
Source: Front Nutr. 2024 Dec 18;11:1506191. doi: 10.3389/fnut.2024.1506191 (PMC11688229; doi:10.3389/fnut.2024.1506191)
Supplement: Supplementary file 2 [file Table_1.DOC]

| **Supplementary Table S1** Basic characteristics between included and excluded participants at baseline. | | | | | |
| --- | --- | --- | --- | --- | --- |
| **Characteristics** | **Overall (n=1,515)** | **Included (n=946)** | **Excluded (n=569)** | ***t*/*χ*2 value** | ***P* value** |
| Age, years | 8.4 (1.5) | 8.0 (1.4) | 8.9 (1.5) | 0.43 | <0.001 |
| Male | 707 (46.6) | 455 (48.1) | 252 (44.2) | 8.04 | 0.157 |
| SBP, mmHg | 106.3 (9.1) | 105.9 (9.1) | 106.9 (9.2) | -0.39 | 0.047 |
| DBP, mmHg | 63.6 (6.6) | 63.4 (6.5) | 63.9 (6.8) | -2.38 | 0.159 |
| GLU, mmol/L | 4.7 (0.5) | 4.6 (0.5) | 4.8 (0.5) | 0.82 | <0.001 |
| HDL-C, mmol/L | 1.5 (0.3) | 1.5 (0.3) | 1.6 (0.3) | 8.44 | 0.302 |
| LDL-C, mmol/L | 2.2 (0.6) | 2.2 (0.6) | 2.2 (0.6) | 8.85 | 0.819 |
| TG, mmol/L | 0.7 (0.3) | 0.7 (0.3) | 0.8 (0.3) | 3.71 | <0.001 |
| TC, mmol/L | 4.1 (0.8) | 4.1 (0.8) | 4.1 (0.8) | -1.15 | 0.290 |
| Insufficient intake of fruits/vegetables | 1230 (81.4) | 784 (82.9) | 446 (78.9) | 1511.98 | 0.067 |
| Frequent intake of carbonated beverages | 93 ( 6.2) | 58 ( 6.1) | 35 ( 6.2) | 7.65 | 1.000 |
| Insufficient PA | 1251 (82.5) | 801 (84.7) | 450 (78.9) | 3.15 | 0.006 |
| Excessive screen time | 79 ( 5.2) | 39 ( 4.1) | 40 ( 7.0) | 0.10 | 0.019 |
| Insufficient sleep | 242 (16.0) | 145 (15.3) | 97 (17.2) | 2.65 | 0.384 |
| Continuous variables are expressed as mean (standard deviation) and categorical variables are expressed as n (%).  SBP, systolic blood pressure; DBP, diastolic blood pressure; GLU, glucose; HDL-C, high-density lipoprotein cholesterol; LDL-C, low-density lipoprotein cholesterol; TG, triglyceride; TC, total cholesterol; PA, physical activity. | | | | | |

| **Supplementary Table S2** The parameters of the Group-based trajectory modeling. | | | | | |
| --- | --- | --- | --- | --- | --- |
| **Outcome** | **Trajectory groups** | **Polynomial degree** | **BIC** | **Percentage of each group (%)** | **Percentage of AvePP over than 0.7 (%)** |
| WC | 1 | one degree | 24683.49 | 100.00 | -- |
|  | 1 | two degree | 24388.72 | 100.00 | -- |
|  | 2 | one degree | 23954.32 | 69.87/30.13 | 65.86/27.27 |
|  | 2 | two degree | 23665.26 | 66.28/33.72 | 63/29.92 |
|  | 3 | one degree | 23844.23 | 59.3/32.98/7.72 | 55.07/30.23/5.39 |
|  | **3** | **two degree** | **23415.64** | **54.23/18.71/27.06** | **50.42/12.68/22.73** |
|  | 4 | one degree | 23763.84 | 27.48/5.29/52.64/14.59 | 18.6/4.12/47.46/10.89 |
|  | 4 | two degree | 23361.96 | 52.54/28.86/10.47/8.14 | 48.31/22.09/7.08/6.34 |
|  | 5 | one degree | 23761.86 | 45.56/18.39/17.97/6.03/12.05 | 39.32/9.51/13/4.76/8.56 |
|  | 5 | two degree | 23362.14 | 46.93/11.63/10.78/22.73/7.93 | 41.23/3.17/7.72/16.07/5.92 |
| WHtR | 1 | one degree | -14067.46 | 100.00 | -- |
|  | 1 | two degree | -14060.74 | 100.00 | -- |
|  | 2 | one degree | -14407.78 | 63.95/36.05 | 58.25/30.44 |
|  | 2 | two degree | -14414.39 | 60.47/39.53 | 55.81/34.88 |
|  | 3 | one degree | -14556.22 | 53.7/10.36/35.94 | 47.99/8.35/32.03 |
|  | **3** | **two degree** | **-14713.25** | **52.85/19.03/28.12** | **47.78/14.8/23.36** |
|  | 4 | one degree | -14537.87 | 10.57/45.77/8.67/34.99 | 8.25/38.69/0/29.49 |
|  | 4 | two degree | -14713.45 | 14.69/51.48/20.72/13.11 | 6.13/46.51/15.12/8.46 |
|  | 5 | one degree | -14554.09 | 10.36/46.09/6.24/18.92/18.39 | 0/39.11/4.12/12.9/11.42 |
|  | 5 | two degree | -14693.24 | 13/10.36/19.56/43.13/13.95 | 4.97/1.37/14.59/37.1/8.88 |
| AvePP, average posterior probability; BIC, Bayesian information criterion; WC, waist circumference; WHtR, waist-to-height ratio. | | | | | |
